# A Non‐Host Pathogen Elicitor Induces Blast Resistance Mediated by OsNAC78‐Pir7b Module in Rice

**DOI:** 10.1111/pce.70500

**Published:** 2026-03-29

**Authors:** Yunjie Xie, Yuling Lai, Xingqian Wu, Xiangzhen Yu, Jingwen Wei, Mengge Chen, Yongsheng Zhu, Liping Chen, Hongguang Xie, Qiuhua Cai, Huaan Xie, Jianfu Zhang

**Affiliations:** ^1^ Rice Research Institute Fujian Academy of Agricultural Sciences Fuzhou China; ^2^ Key Laboratory of Germplasm Innovation and Molecular Breeding of Hybrid Rice for South China Ministry of Agriculture and Affairs Beijing P.R. China; ^3^ Incubator of National Key Laboratory of Germplasm Innovation and Molecular Breeding between Fujian and Ministry of Sciences and Technology/Fuzhou Branch National Rice Improvement Center of China/Fujian Engineering Laboratory of Crop Molecular Breeding/Fujian Key Laboratory of Rice Molecular Breeding Fuzhou China; ^4^ College of Plant Protection Fujian Agriculture and Forestry University Fuzhou China

**Keywords:** blast disease, immunity, NAC family, non‐host pathogen, Pir7b

## Abstract

Plants exhibit broad‐spectrum and persistent resistance induced by non‐host pathogens. Previous studies have found that syringolin A secreted by *Pseudomonas syringae* pv. *syringae* can activate the expression of defense‐related gene *Pir7b* in non‐host rice, but the underlying mechanism remains ambiguous. In this study, we found that OsNAC78, a transcription factor upstream of Pir7b, is possible to participate in the immune pathway. First, the OsNAC78 transgenic plants were inoculated with *Magnaporthe oryzae*, showing that OsNAC78 is a positive regulator for blast resistance. Meanwhile, the transgenic lines overexpressing or knocking out *Pir7b* were constructed and evaluated for resistance, displaying that Pir7b confers blast resistance. Subsequently, OsNAC78‐Pir7b module is activated by enhancing activity of *OsNAC78* and *Pir7b* promoters under the syringolin A treatment. The transcriptional activation of *Pir7b* is blocked in the absence of OsNAC78, indicating that OsNAC78 is a key regulator of *Pir7b* expression. Further, it was found that syringolin A can activate the strongest resistance in the wild type, while the weakest in the *Osnac78*/*pir7b* mutant, suggesting that syringolin A induces rice immunity through the OsNAC78‐Pir7b module. Overall, we discovered that syringolin A secreted by non‐host pathogens can induce the expression of the transcription factor OsNAC78 in rice, subsequently upregulate the transcription of the downstream gene *Pir7b*, and ultimately trigger blast resistance by ROS accumulation. This study reveals how the secretion of non‐host pathogen stimulates plant immune system, providing new insights for plant disease control.

## Summary statement

Non‐host pathogens often induce plant resistance, but the underlying mechanisms remain ambiguous. This study reveals that syringine A, secreted by the non‐host pathogen P. syringae, is an elicitor that can trigger resistance in rice. Upon syringine A is perceived by rice receptors, the transcription factor OsNAC78 is activated through signal transduction, thereby enhancing the transcription of the downstream target *Pir7b*, a defense‐related gene. This process influences the accumulation of ROS, ultimately inducing resistance to rice blast disease. The research provides an example of how non‐host pathogens can induce rice resistance.

## 1 Introduction

Plants are constantly exposed to various microbial threats in their natural environment. Their robust innate immune system provides durable resistance against the majority of pathogens. As the first layer of immune defense, cell surface‐localized pattern recognition receptors (PRRs) in plants specifically perceive the conserved pathogen‐associated molecular patterns (PAMPs) of pathogens and activate the immune response, called PAMP‐triggered immunity (PTI; Boller and He 2009; Couto and Zipfel 2016). The typical PAMPs include bacterial flagellin, fungal chitin, oomycete elicitin, and viral capsid protein, etc (Boutrot and Zipfel 2017). PRR‐PAMP recognition triggers the conversion of transmembrane signals. The cyclic nucleotide‐gated channel (CNGC) proteins assemble to form ion channels that promote calcium ion (Ca2+) influx; The NADPH oxidase RBOHD, after phosphorylation modification, produces reactive oxygen species (ROS) bursts (Kadota et al. 2014; Tian et al. 2019; Rui et al. 2025). Collaboratively, these processes form an early signaling hub. The downstream mitogen‐activated protein kinase (MAPK) phosphorylation cascade further transmits signals to the nucleus, inducing key factors such as WRKY/MYC2/OsEDC4 to regulate the expression of defense genes (Li et al. 2012; Yu et al. 2024a; Lu et al. 2024). This ultimately leads to callose deposition in the cell wall, biosynthesis of phytoalexins, and accumulation of pathogenesis‐related proteins.

The recognition of PAMPs‐PRRs is not limited to the interactions between pathogens and hosts; conserved PAMPs from non‐host pathogens also be perceived by host plants, triggering HR responses. For example, the oomycete Phytophthora infestans, infecting potato and tomato, secretes multiple RXLR effectors that can be recognized by non‐host pepper, inducing an immune response (Lee et al. 2014). Similarly, it has been found that the effector PcAvr3a‐like secreted by Phytophthora capsici can be perceived by Nicotiana species, mediating nonhost resistance (Vega‐Arreguín et al. 2014). The syringolin A secreted by nonhost pathogen *Pseudomonas syringae* pv. *Syringae*, as a novel peptide elicitor, can be recognized by rice, activating the expression of the defense‐related gene *Pir7b*, which encodes an esterase (Reimmann et al. 1995; Hassa et al. 2000). In previous reports, Pir7b might have the sign of inducing resistance to rice blast (Wäspi et al. 2007). However, there is currently no direct evidence suggesting that Pir7b is involved in regulating the resistance to rice blast. Moreover, the mechanism by which Pir7b expression is regulated with syringolin A‐mediated activation remains unclear.

The NAC (NAM, ATAF1/2, and CUC2) family is a group of plant‐specific transcription factors (TFs), with a conserved DNA‐binding domain at its N‐terminal and a highly variable transcriptional regulatory domain at its C‐terminal (Jin et al. 2017; Mohanta et al. 2020; Chang et al. 2024;). The NAC TFs often act crucial roles in plant growth and development, as well as in responding to abiotic and biological stresses (Olsen et al. 2005; Han et al. 2023; Xiong et al. 2025). During the root development of rice, OsNAC2 regulates the division of root cells as a key upstream integrator of auxin and cytokinin signals (Mao et al. 2020a). NACs functions on fruit yield and quality by determining the inflorescence of the shoot apical meristem and controlling fruit ripening (Liu et al. 2023). Under drought stress, SNAC1 and OsERF103 synergistically regulate the expression of *OsbZIP23*, as a key player of ABA signaling pathway and drought response to resist water‐scarce environment (Xiang et al. 2008; Li et al. 2019; Yang et al. 2024); a drought responsive gene *TaSNAC8‐6A* in wheat germplasm pool impvoves drought tolerance by enhancing lateral root development (Mao et al. 2020b). In recent years, there have been an increasing number of reports on which NAC family is involved in immune regulation. Under pathogen stress, the NAC transcription factor SlNAP1 in tomato positively regulates defense resistance against two widespread bacterial diseases (Wang et al. 2020). In rice, MNAC3‐centered regulatory network acts a negative role in immunity. The protein phosphatase OsPP2C41 dephosphorylates MNAC3, weakening transcriptional activation of downstream immune‐negative genes (Wang et al. 2024). Interestingly, NAC90 interacting with NAC61 and NAC36 in Arabidopsis form a NAC triad to control immune resistance by negatively regulate N‐hydroxy pipecolic acid biosynthesis (Cai et al. 2024). Overall, the NAC family unique to plants plays an irreplaceable role in various biological processes.

In our previous research, we utilized 10 × Genomics' single‐cell RNA sequencing method to predict the downstream targets of the transcription factor OsNAC78 in rice, ultimately capturing two target genes, respectively *OsGSTu37* encoding a ROS‐scavenging enzyme and *Pir7b* encoding an esterase (Xie et al. 2020). We have clarified the mechanism by which OsNACIP6/OsNAC78‐OsGSTU37 module regulates drought tolerance in rice (Yu et al. 2024b). In this study, we found that OsNAC78‐Pir7b module was induced the syringolin A secreted by nonhost pathogen to enhance rice blast resistance. This result clarifies that the secretions of non‐host pathogen can stimulate the immune system of plant, providing a novel idea for control of plant diseases.

## 2 Materials and Methods

### 2.1 Plant Materials and Growth Conditions

Rice indica cultivar ‘75‐1‐127’ and japonica cultivar ‘Nipponbare’ (Nip) were used as wild type (WT) and background materials in this study. The *Pir7b* knockout mutants were generated using CRISPR‐Cas9, and *Pir7b* was placed under the control of the ubiquitin promoter to generate overexpression lines. Plants were grown at the experimental stations in Fujian or Hainan Province for seed production, and transgenic seedlings were grown in a greenhouse set at 28°C and 70% relative humidity with a 16‐h day/8‐h night photoperiod.

### 2.2 Vector Construction and Generation of Transgenic Rice Plants

Vector construction and rice transformation CRISPR‐Cas9 vectors were constructed by following the protocol reported previously (Xing et al. 2014). *Pir7b* targeting sequence was generated by referring to the website (http://www.genome.arizona.edu/crispr/CRISPRsearch.html). pCBC‐MT1T2 was used as the template for PCR amplification with the specific target primers. The products were inserted into the pHUE411 vector to generate CRISPR‐Cas9 vectors. The coding region of *Pir7b* cloned from *Nip* was amplified using specific primers and inserted into the pCUbi1390 vector harboring the ubiquitin promoter to generate the overexpression vector. The vectors were introduced into *Agrobacterium tumefaciens* stain EHA105 and then transferred into embryogenic calli via *Agrobacterium*‐mediated transformation. T2 positive plants were obtained and used in this study.

### 2.3 *M. oryzae* Inoculation Assay and Field Trial

To evaluate rice blast resistance in plants, spray and punch inoculations were used. For the spray inoculation, 2‐week‐old seedlings were sprayed with a spore suspension (1 × 10^5^ spores/mL) of *M. oryzae* isolates as described previously (Qu et al. 2006). The phenotypes were observed at 7 dpi. For the punch‐inoculation, the leaves of 4‐week‐old rice seedlings were inoculated with *M. oryzae* as described previously (Li et al. 2017). The leaves were incubated on 6‐benzylaminopurine medium (5 g of agar, 2.5 mg of 6‐benzylaminopurine per liter), and 5 μL spore suspension (1 × 10^5^ spores/mL) was placed on each punch spot. At 7 dpi, the lesion lengths were measured using vernier calipers, and the relative fungal biomass of *M. oryzae* was evaluated by normalizing the threshold cycle value (CT) of *M. oryzae Pot2* DNA against the CT of rice genomic ubiquitin DNA from DNA‐based real‐time PCR (Park et al. 2012). All the inoculation experiments were repeated three times independently. Field trials were performed in a natural rice blast nursery (Longyan, Fujian province, China) having a high selection pressure of rice blast.

### 2.4 RNA Extraction and qRT‐PCR

Total RNA was extracted using TRIzol reagent (Transgen Biotech, Beijing, China) and reversed transcribed using RNA reversetranscription kit with gDNA Remover (Toyobo, Osaka, Japan) according to the manufacturer's instructions. qRT‐PCR was performed using a FastStart Universal SYBR Green Master (ROX) Kit (Roche, Basel, Switzerland). The reaction solution contained 10 µL of SYBR Green Mix, 8.25 µL of sterile water, 0.375 µL of each primer, and 1 µL of cDNA. The PCR program consisted of an initial denaturation step 95°C for 10 min, followed by 40 cycles of 95°C for 10 s and 60°C for 30 s. The ubiquitin gene was used as an internal control, and the relative expression levels were obtained using the ΔΔCt method. All experiments were repeated 3 times biologically and primers used in the assay are listed in Supporting Information S2: Table 1.

### 2.5 Protoplast Isolation and Transfection

Rice protoplast isolation was performed according to Zhang et al. (2011). Briefly, 2‐week‐old rice seedlings were cut into 0.5‐mm strips and immediately transferred into the enzyme solution [0.6 M d‐mannitol, 10 mM MES, 1.5% Cellulase R‐10, 0.75% Macerozyme R‐10, 0.1% BSA, 3.4 mM CaCl2 at pH 5.7, Sigma], for 3 h shaking with 70 rpm in darkness at 28°C. After pouring off the enzyme solution, the strips were washed using W5 solution (2 mM MES, 154 mM NaCl, 5 mM KCl, and 125 mM CaCl2 at pH 5.7; Sigma‐Aldrich, St. Louis, MO, USA) and filtered with an 80‐μm mesh. The protoplasts were collected by centrifuging for 3 min at 300×g, washed with the W5 solution, after which the supernatant was removed and resuspended in MMG solution (0.6 M D‐mannitol, 15 mM MgCl2, 4 mM MES at pH 5.7; Sigma‐Aldrich). Protoplasts transfection was performed using the PEG‐mediated method as described by Yoo et al. (2007). Briefly, each sample containing 5 µg of plasmids and 100 µL of protoplasts was mixed and added the same volume of PEG solution [0.6 M d‐mannitol, 100 mM CaCl2, and 40% PEG4000; Sigma‐Aldrich]. The mixture was incubated at 28°C for 15 min, and W5 solution was added to stop the reaction. Protoplasts were collected by centrifuging and then resuspended in W5 solution at 28°C in darkness.

### 2.6 Subcellular Localization

The full‐length CDS of Pir7b was amplified by PCR and respectively inserted into pRTVcGFP driven by ubiquitin promoter. The recombinant plasmids were transiently expressed in rice protoplasts with a nuclear marker. After incubation at 28°C for 16 h in darkness, a confocal microscope (Leica) was used to observe the fluorescence in protoplasts for subcellular localization.

### 2.7 EMSA Analysis

The full‐length CDS of *OsNAC78* was inserted into pMAL‐c5X, generating MBP‐NAC78. Expression of MBP and MBP‐NAC78 in *Escherichia coli Rosetta* (*DE3*) was induced by 0.1 mM isopropyl β‐d‐thiogalactoside at 16°C for 16 h. For the EMSA assay, the double‐stranded Cy5.5‐labeled probes and mutant double‐stranded Cy5.5‐labeled probes used in this assay were synthesized (Biosun, Shanghai, China), and unlabeled probes acting as competitors were also synthesized (Biosun). All probe sequences are listed in Supporting Information S2: Table 1. The EMSA assay was performed using an EMSA/Gel‐Shift Kit (Beyotime, Shanghai, China) following the manufacturer's protocol. In brief, 2 mg of purified MBP‐NAC78 protein was added to the binding reaction and incubated at 25°C for 20 min in a thermal cycler (BioRad, Hercules, CA, USA). The mixture was separated on a 4% polyacrylamide gel in 0.5×Tris borate‐EDTA buffer, and the gel images were visualized using the Odyssey Infrared Imaging System (LI‐COR, Lincoln, NE, USA).

### 2.8 Dual‐Luciferase Reporter Assay in Rice Protoplasts

The promoters of *OsNAC78* and *Pir7b* were amplified and inserted independently into the pGreen II 0800 vector, generating pro*NAC78*::*LUC* and pro*Pir7b*::*LUC*, respectively. The mini35S promoter was used as a negative control. Then, the recombinant vectors were independently transfected into protoplasts. The transfected protoplasts were cultured with or without 500 nM syringolin A treatment for 24 h at 28°C in darkness. The LUC and REN activities were separately measured using a Dual Luciferase Reporter Assay Kit (Vazyme, Nanjing, China). The relative luciferase activities are represented by LUC/REN ratios. All the experiments were repeated biologically three times.

### 2.9 Syringolin A Treatment

To investigate the induction of *OsNAC78* or *Pir7b* expression by syringolin A, experiments were conducted using rice protoplast treatment, rice plant treatment, and dual‐luciferase reporter assay. For syringolin A‐treated rice protoplast experiment, *OsNAC78* and *Pir7b* expression level in 75‐1‐127 rice protoplasts at a concentration of 500 nM syringolin A were measured at different time points by qRT‐PCR. For syringolin A‐treated rice plant experiment, 20 μM syringolin A was sprayed uniformly on the leaves of 75‐1‐127, and samples were collected at different time points to extract total RNA, and the expression levels of *OsNAC78* and *Pir7b* were detected by qRT‐PCR. For the dual‐luciferase reporter assay, combination constructs were transfected into 75‐1‐127 or *Osnac78* mutant rice protoplasts. The min35S was used as a control. The cultures were treated with 500 nM syringolin A, after 24 h of dark incubation at 28°C, luciferase assays were performed. The relative LUC/REN ratio indicated the transcription activities of min35S, *OsNAC78*, and *Pir7b* promoters under syringolin A treatment or without syringolin A treatment.

### 2.10 Non‐Target Metabolomics Analysis

Non‐target metabolomics were performed on *pir7b* mutants and wild‐type (Nip) varieties after styring with syringolin A. This work was completed by MetWare Company (Wuhan, China). Accurately weigh 30 mg of sample powder using an electronic balance (MS105DΜ) and add 1500 μL of −20°C pre‐cooled 70% methanolic aqueous internal standard extract. Vortex once every 30 min for 30 s, for a total of 6 times. After centrifugation (rotation speed 12000 rpm, 3 min), the supernatant was aspirated, and the sample was filtered through a microporous membrane (0.22 μm pore size) and stored in the injection vial for UPLC‐MS/MS analysis. All samples were acquired by the LC‐MS system followed machine orders. The analytical conditions were as follows, UPLC: column, Waters ACQUITY UPLC HSS T3 1.8 µm, 2.1 mm * 100 mm; column temperature, 40°C; flow rate, 0.40 mL/min; injection volume, 4 μL; solvent system, water (0.1% formic acid): acetonitrile (0.1% formic acid). All the methods alternated between full scan MS and data dependent MSn scans using dynamic exclusion. MS analyses were carried out using electrospray ionization in the positive ion mode and negative ion mode using full scan analysis over m/z 84‐1250 at 35000 resolution.

Raw mass spectrometry data were converted to mzML format using ProteoWizard, followed by peak extraction, alignment, and retention time correction via the XCMS software. Metabolite abundance data underwent UV (unit variance scaling) processing. Using the MetaboAnalystR package's OPLSR. Anal function in R software, an orthogonal partial least squares discriminant analysis (OPLS‐DA) score plot was generated to visualize differences between groups. Differential metabolites were screened based on the criteria of |log2Fold Change | ≥ 2 or |log2Fold Change | ≤ 0.5 and Variable Importance Projection (VIP) ≥ 1, and annotated to the KEGG database.

## 3 Results

### 3.1 OsNAC78 Positively Regulates Resistance to Rice Blast

**Figure 1:**
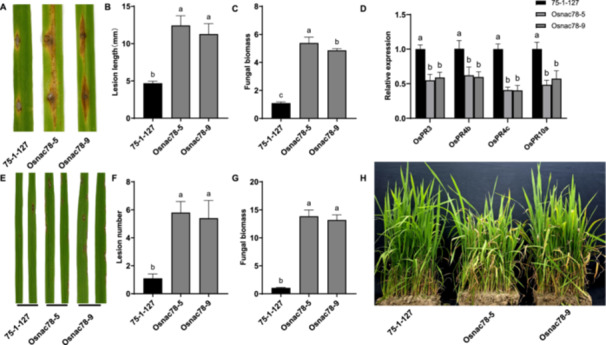
OsNAC78 positively regulates resistance to *M. oryzae* in 75‐1‐127. (A) Photographs of *Osnac78* mutants at 7 days post punch‐inoculation with the 94‐6‐1Q isolate. (B) Vertical lengths of lesions induced by 94‐6‐1Q isolate were measured. Values represent the mean ± SE (*n* = 10, *n* means biological replicate). Statistical significance was determined by one‐way ANOVA *(p* < 0.05). (C) The fungal biomass of the 94‐6‐1Q isolate was calculated by qRT‐PCR using *MoPot2*/*OsUbq5*. Values represent the mean ± SE (*n* = 3, *n* means biological replicate). Statistical significance was determined by one‐way ANOVA (*p* < 0.05). (D) The expression levels of pathogenesis‐related genes in *Osnac78* mutants inoculated with 94‐6‐1Q isolate were detected by qRT‐PCR. *OsUbq5* was used for the normalization. Values represent the mean ± SE (*n* = 3, *n* means biological replicate). Statistical significance was determined by one‐way ANOVA (*p* < 0.05). (E) Photographs of *Osnac78* mutants at 7 days post spray‐inoculation with the DWNH20210243 isolate. (F) The lesion numbers after the DWNH20210243 isolate treatment were analyzed. Values represent the mean ± SE (*n* = 10, *n* means biological replicate). Statistical significance was determined by one‐way ANOVA (*p* < 0.05). (G) The fungal biomass of the DWNH20210243 isolate in *Osnac78* mutants were calculated by qRT‐PCR using *MoPot2*/*OsUbq5*. Values represent the mean ± SE (*n* = 3, *n* means biological replicate). Statistical significance was determined by one‐way ANOVA (*p* < 0.05). (H) Photographs showing the field blast resistance of *Osnac78* mutants in the natural rice blast nursery. Red arrows represent lesions induced by *M. oryzae*. Enlarged images of the boxed areas are shown on the right.

Based on 10 × Genomics' single‐cell RNA sequencing analysis that the binding target of OsNAC78 was predicted to be defense‐related gene *Pir7b* in rice (Luo et al. 2008; Xie et al. 2020), thus we hypothesize that OsNAC78 may possess functions related to disease resistance. The blast resistance evaluation was conducted on *Osnac78* mutants. After punch‐inoculation with 94‐6‐1Q isolate, *Osnac78* mutants displayed larger disease lesion compared with wild type (WT) variety ‘75‐1‐127’ at 7 days post inoculation; and the fungal biomass of *Osnac78* mutants was also markedly higher than that of WT (Figure 1A–C). The expression levels of pathogenesis‐related genes in *Osnac78* mutants inoculated with 94‐6‐1Q isolate for 36 h were detected by qRT‐PCR. The results showed that the expression levels of pathogenesis‐related genes was significantly decreased in *Osnac78* mutants than that in WT (Figure 1D). The spray‐inoculation assays yielded consistent results, with *Osnac78* mutants exhibiting more lesions compared to WT (Figure 1E–G). To further assess the field resistance of OsNAC78, we conducted natural field trials in high‐incidence area for blast disease. Field observation revealed that *Osnac78* mutants developed noticeable disease lesions, indicating blast resistance of plant was significantly weakened under the absence of OsNAC78 (Figure 1H; Supporting Information S1: Figure 1A,B). Moreover, the punch‐inoculation assays showed that the blast resistance of rice plants was significantly enhanced after overexpression of OsNAC78 (Supporting Information S1: Figure 1C–E). These findings demonstrate that OsNAC78 in rice positively regulates resistance to blast disease.

### 3.2 The Transcription Factor OsNAC78 Binds to the Promoter of Pir7b

**Figure 2:**
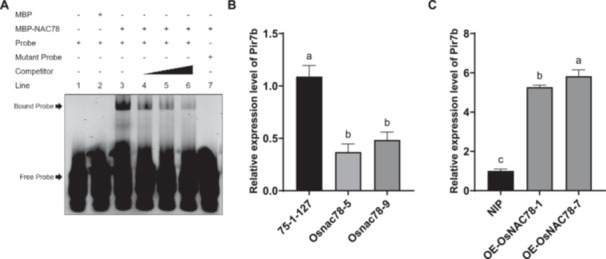
The transcription factor OsNAC78 binds to the promoter of *Pir7b*. (A) The interaction between OsNAC78 and a CY5.5‐labeled probe containing one CGTG motif in the *Pir7b* promoter was shown using EMSA. Unlabeled probe was used as a competitor. The triangle denotes an increasing dose. The CY5.5‐labeled probe from CGTG motif mutating into CaaG motif was used as a mutant probe. MBP was used as a control. (B) *Pir7b* expression level in different *Osnac78* mutants plants and WT (75‐1‐127) measured with qRT‐PCR. The expression level in the 75‐1‐127 is set to 1.0 and error bars represent the SE of 3 independent experiments. Statistical significance was determined by one‐way ANOVA (*p* < 0.05). (C) The Pir7b expression level in different OsNAC78 over‐expression plants and WT (NIP) measured with RT‐qPCR. The expression level in the NIP is set to 1.0 and error bars represent the SE of three independent experiments. Statistical significance was determined by one‐way ANOVA (*p* < 0.05).

To further verify that transcription factor OsNAC78 can bind to the promoter of Pir7b, we performed an electrophoretic mobility shift assay (EMSA) assay. The results indicated that OsNAC78 bound to the probe containing the CGTG motif in the OsPir7b promoter; with the addition of competing probe in an increasing dose, this binding gradually weakened; moreover, when the CGTG motif is mutated into the CaaG motif, this binding was completely abolished (Figure 2A). Following, the transcriptional expression of Pir7b was detected by qRT‐PCR under the absence of transcription factor OsNAC78. The results showed that the expression levels of *Pir7b* in *Osnac78* mutants were significantly lower than that in WT (Figure 2B). These findings demonstrate that Pir7b is regulated as a downstream target by OsNAC78.

### 3.3 Pir7b Confers Blast Resistance in Rice by Regulating ROS Accumulation

**Figure 3:**
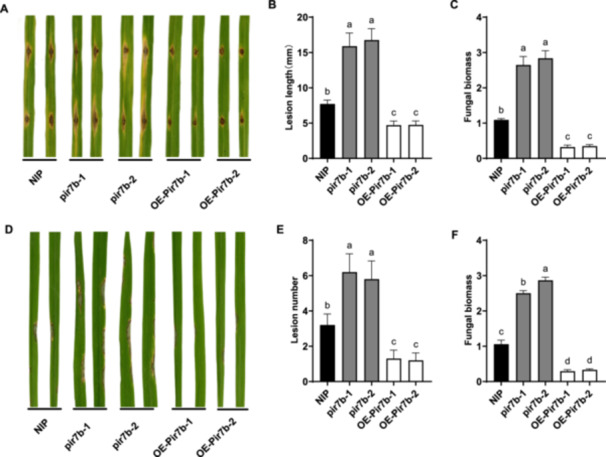
Pir7b positively regulates resistance to *M. oryzae* in Nipponbare. (A) Photographs of *Pir7b* transgenic lines at 7 days post punch‐inoculation with the Guy11 isolate. (B) Vertical lengths of lesions induced by Guy11 isolate were measured. Values represent the mean ± SE (*n* = 10, *n* means biological replicate). Statistical significance was determined by one‐way ANOVA (*p* < 0.05). (C) The fungal biomass of the Guy11 isolate was calculated by qRT‐PCR using *MoPot2*/*OsUbq5*. Values represent the mean ± SE (*n* = 3, *n* means biological replicate). Statistical significance was determined by one‐way ANOVA (*p* < 0.05). (D) Photographs of *Pir7b* transgenic lines at 7 days post spray‐inoculation with the Guy11 isolate. (E) The lesion numbers after the Guy11 isolate treatment were analyzed. Values represent the mean ± SE (*n* = 10, *n* means biological replicate). Statistical significance was determined by one‐way ANOVA (*p* < 0.05). (F) The fungal biomass of the Guy11 isolate in *Pir7b* transgenic lines were calculated by qRT‐PCR using *MoPot2*/*OsUbq5*. Values represent the mean ± SE (*n* = 3, *n* means biological replicate). Statistical significance was determined by one‐way ANOVA (*p* < 0.05). [Color figure can be viewed at wileyonlinelibrary.com]

To verify that Pir7b is directly associated with blast resistance, we used the rice cultivar ‘Nipponbare’ (Nip) as the transformation background material to produce homozygous *pir7b‐1* and *pir7b‐2* mutants, which respectively harbor a 1‐bp deletion and 2‐bp deletion (Supporting Information S1: Figure 2A), and two independent *Pir7b*‐overexpressing (OE) transgenic lines (Supporting Information S1: Figure 2B). After punch‐ and spray‐inoculation with Guy11 isolate, *pir7b* mutants exhibited significantly larger relative lesion areas and higher fungal biomass than WT; Conversely, OE‐*Pir7b* lines showed significantly smaller relative lesion areas and lower fungal biomass than WT (Figure 3). The resistance phenotypes of *Pir7b* transgenic lines indicate that Pir7b in rice positively regulates resistance to blast disease. To further elucidate the regulatory mechanism, we measured ROS‐related parameters in *Pir7b* transgenic lines. The results showed that compared to WT, *pir7b* mutants exhibited lower ROS accumulation, while OE‐*Pir7b* lines displayed higher ROS accumulation (Supporting Information S1: Figure 3). These findings suggest that Pir7b positively regulates ROS accumulation to confer blast resistance.

### 3.4 The Expression Pattern of Pir7b

**Figure 4:**
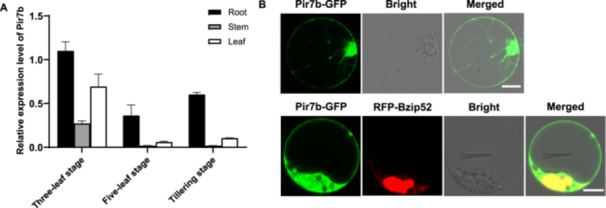
Expression patterns of Pir7b. (A) The relative expression level of *Pir7b* in Nip at different growth stages measured with qRT‐PCR. The error bars represent the SE of 3 independent experiments. (B) Pir7b protein was expressed alone in rice protoplasts, top. Pir7b protein was co‐expressed with nuclear maker RFP‐Bzip52 in rice protoplasts. Genes were driven by the ubiquitin promoter. Scale bars, 5 μm. [Color figure can be viewed at wileyonlinelibrary.com]

The coding sequence (CDS) of the *Pir7b* gene measures 807 bp, encoding a protein containing 268 a.a. Comparative analysis with proteins from *Oryza sativa* Japonica Group, *Triticum aestivum*, *Zea mays* and *Arabidopsis thaliana* revealed several homologous genes. These homologs were used to construct a phylogenetic tree, where XP_044337741.1 (LOC123059179) showed the nearest genetic distance to Pir7b in the phylogenetic tree (Supporting Information S1: Figure 4). To investigate the expression levels of *Pir7b* in various tissues of rice at different growth stages, we detected the expression of *Pir7b* under normal conditions by qRT‐PCR. Total RNA respectively isolated from root, stem, and leaf at three‐leaf stage, five‐leaf stage, and tillgering stage in Nip. The results showed that the expression level of *Pir7b* is highest in the root at the three growth stages, followed by the leave and stem (Figure 4A). Meanwhile, to visualize the cellular localization of Pir7b protein in rice, a subcellular expression vector, pRTVcGFP‐Pir7b, carrying a green fluorescent protein GFP tag was constructed. This vector was introduced into rice protoplasts for expression and analyzed using fluorescence confocal microscopy. Partial colocalization between Pir7b‐GFP and the nuclear marker RFP‐Bzip52, with areas of non‐colocalization, was observed, indicating that Pir7b protein is distributed in both the nucleus and cytoplasm (Figure 4B).

### 3.5 Syringolin A Induces the Expression of OsNAC78 and Pir7b

**Figure 5:**
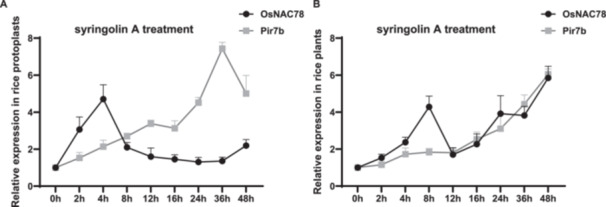
Syringolin A induces the expression of *OsNAC78* and *Pir7b*. (A) *OsNAC78* and *Pir7b* expression in rice protoplasts at a concentration of 500 nM syringolin A measured with qRT‐PCR. The expression level in the 0 h is set to 1.0 and error bars represent the SE of 3 independent experiments. (B) *OsNAC78* and *Pir7b* expression in rice plants at a concentration of 20 μM syringolin A measured with qRT‐PCR. The expression level in the 0 h is set to 1.0 and error bars represent the SE of 3 independent experiments.

To verify whether the expression of transcription factor *OsNAC78* and its downstream target *Pir7b* is induced by *M. oryzae*, WT plants were inoculated with *M. oryzae* and ddH_2_O as a control. Total RNA was extracted from leaves harvested at different time points, and qRT‐PCR analysis showed that neither *OsNAC78* nor *Pir7b* was significantly induced by *M. oryzae* (Supporting Information S1: Figure 5). Given prior reports that *Pir7b* expression is induced by syringolin A secreted by *P. syringae*, we hypothesize this process may be directly linked to the upstream transcription factor OsNAC78. The rice protoplasts were prepared and treated with 500 nM syringolin A. Total RNA was extracted from samples at different time points, and qRT‐PCR was used to detect *Pir7b* and *OsNAC78* gene expression. The results indicate that both the transcription factor *OsNAC78* and its downstream target *Pir7b* are induced by syringolin A. *OsNAC78* expression began to rise at 2 h post‐treatment, peaking at 4 h, and *Pir7b* expression was induced starting at 2 h and reached its peaking at 36 h (Figure 5A). To further validate, 20 μM syringolin A was uniformly sprayed onto leaves, harvested at various time points. The qRT‐PCR results displayed that both *OsNAC78* and *Pir7b* expression levels were induced starting at 2 h and peaked at 48 h post‐treatment (Figure 5B). These findings indicate that the OsNAC78‐Pir7b module is activated by syringolin A secreted by *P. syringae*.

### 3.6 OsNAC78 as a Key Factor Participates in the Syringolin A‐Mediated Pir7b Expression Pathway

**Figure 6:**
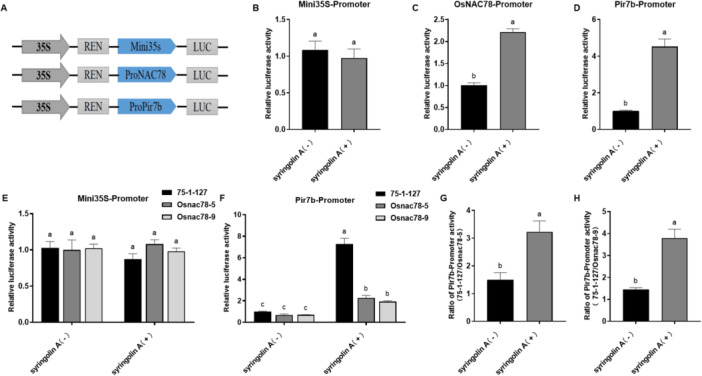
Syringolin A mainly induces the expression of *Pir7b* through OsNAC78. (A) Schematic graph of vectors used for the dual‐luciferase reporter assays. (B–F) The dual‐luciferase reporter assays in rice protoplasts. The relative luciferase activity from LUC/REN ratio indicated the relative transcription activities of min35S, *OsNAC78*, and *Pir7b* promoters under syringolin A treatment or without syringolin A treatment. After 24 h incubation, rice protoplasts were collected and detected. The min35S was used as a control. Data present the mean ± se (*n* = 3). Statistical significance was determined by one‐way ANOVA (*p* < 0.05). (E and F) The dual‐luciferase reporter assays in WT (75‐1‐127) and *Osnac78* protoplasts. (G, H) The ratio of Pir7b‐promoter activity in 75‐1‐127 and Osnac78 protoplasts under syringolin A treatment or without syringolin A treatment. Data present the mean ± se (*n* = 3). [Color figure can be viewed at wileyonlinelibrary.com]

To further validate that *Pir7b* expression is induced by syringolin A, a dual‐luciferase reporter assay was performed. The rice protoplasts from WT plants were prepared and separately transfected with the vectors pro*NAC78*::*LUC* and pro*Pir7b*::*LUC*, which drive firefly luciferase expression under the *OsNAC78* and *Pir7b* promoter, respectively (Figure 6A). These were then treated with 500 nM syringolin A, with the mini35S promoter serving as a control. After incubation, luciferase assays were performed, and data were analyzed to compare the Luc/Ren ratios across groups, revealing the effects of syringolin A treatment on target gene promoters. The results showed that the mini35S promoter exhibited no sensitivity to syringolin A, whereas transcription activity of the *OsNAC78* and *Pir7b* promoters increased to varying degrees under syringolin A treatment (Figure 6B–D). This indicates that OsNAC78 and Pir7b respond to syringolin A‐induced signal transduction. Subsequently, the rice protoplasts from WT (75‐1‐127) and two *Osnac78* mutants were individually transformed with the vector pro*Pir7b*::*LUC* and treated with 500 nM syringolin A, using the mini35S promoter as a control. The results displayed that syringolin A induced enhanced transcription activity of the *Pir7b* promoter in both 75‐1‐127 and *Osnac78* protoplasts, but had no effect on the transcription activity of the mini35S promoter in both 75‐1‐127 and *Osnac78* protoplasts (Figure 6E, F). Moreover, the ratio of Pir7b‐promoter activity in 75‐1‐127 and *Osnac78* protoplasts under syringolin A treatment, is significantly higher than that without treatment of syringolin A (Figure 6G,H). This suggests that the syringolin A‐induced Pir7b activation in *Osnac78* mutants is attenuated compared to that in 75‐1‐127. These data demonstrate that syringolin A cannot absolutely induce *Pir7b* expression in absence of OsNAC78 in rice. Therefore, we speculate that OsNAC78 functions as a key transcription factor in the syringolin A‐mediated Pir7b expression pathway.

### 3.7 The Syringolin A Elicits Resistance to Rice Blast Through the OsNAC78‐Pir7b Module

**Figure 7:**
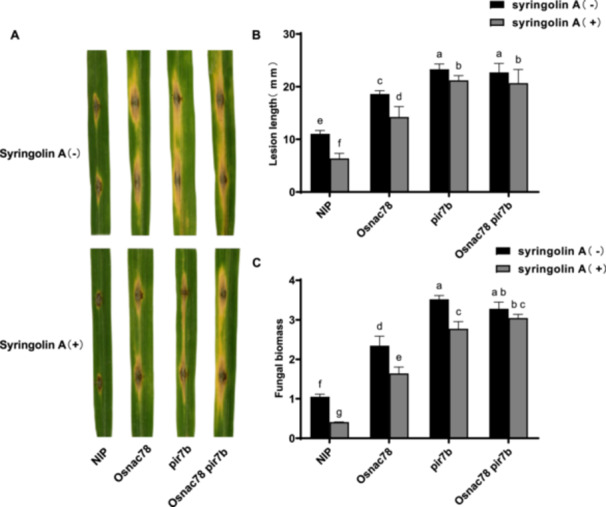
Syringolin A activates blast resistance through the OsNAC78‐Pir7b module. (A) Photographs of *Osnac78*, *pir7b* and *Osnac78*/*pir7b* mutants at 7 days post punch‐inoculation with the Guy11 isolate. (B) Vertical lengths of lesions induced by Guy11 isolate were measured. Values represent the mean ± SE (*n* = 10, *n* means biological replicate). Statistical significance was determined by one‐way ANOVA (*p* < 0.05). (C) The fungal biomass of the Guy11 isolate was calculated by qRT‐PCR using *MoPot2*/*OsUbq5*. Values represent the mean ± SE (*n* = 3, *n* means biological replicate). Statistical significance was determined by one‐way ANOVA (*p* < 0.05). [Color figure can be viewed at wileyonlinelibrary.com]

To investigate whether syringolin A directly induces blast resistance via the OsNAC78‐Pir7b module, the leaves of WT and *Osnac78*, *pir7b*, and *Osnac78*/*Pir7b* mutants in vitro were inoculated with *M. oryzae* using media containing 5 μM syringolin A or without syringolin A. It was found that the WT and *Osnac78*, *pir7b*, *Osnac78*/*Pir7b* mutants under syringolin A treatment all displayed more resistant to *M. oryzae* than without syringolin A. Importantly, syringolin A maximally activated WT plant resistance to blast fungus, however, the double knockout of *OsNAC78* and *Pir7b* significantly weakened syringolin A‐induced blast resistance (Figure 7). This indicates that the OsNAC78‐Pir7b module plays a crucial role in the syringolin A‐mediated rice immune pathway.

### 3.8 Untargeted Metabolites in *pir7b* Mutants and Wild‐Type Varieties after Spraying Syringolin A

**Figure 8:**
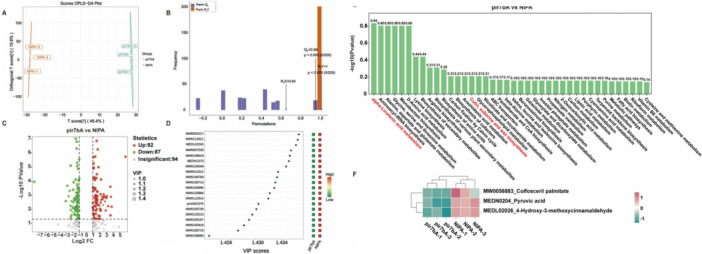
Non‐targeted metabolomics analysis of pir7bA and NIPA. (A) The OPLS‐DA score plot for pir7bA_vs_NIPA. The horizontal axis direction indicated differences between groups; the vertical axis represents orthogonal principal components, with its direction revealing differences within groups. (B) OPLS‐DA validation plot. R2X and R2Y denote the explanatory power of the constructed model for the X and Y matrices, respectively, while Q2 indicates the model's predictive capability. The closer these three metrics are to 1, the more stable and reliable the model is. A Q2 value greater than 0.5 indicates an effective model, while a Q2 value greater than 0.9 signifies an excellent model. (C) Volcano plot of differentially expressed metabolites between pir7bA and NIPA. Green dots represent downregulated metabolites, red dots represent upregulated metabolites, and gray dots indicate metabolites detected but not significantly different. (D) VIP Value Plot + Heatmap (VIP > 1) displayed only the top 20 differentially expressed metabolites. (E) Bar chart showing KEGG pathway enrichment significance for differential metabolites, displaying the top 40 pathways. Pathways annotated in red were those associated with ROS activity. (F) Heatmap of the contents of MW0056883_Colfosceril palmitate, MEDN0204_Pyruvic acid, and MEDL02026_4‐Hydroxy‐3‐methoxycinnamaldehyde. [Color figure can be viewed at wileyonlinelibrary.com]

The *pir7b* mutants and wild‐type (Nip) varieties were sprayed with syringolin A and designated as pir7bA and NIPA, respectively, for untargeted metabolomics analysis. To elucidate metabolic changes in pir7bA and NIPA, orthogonal partial least squares discriminant analysis (OPLS‐DA) was employed to amplify intergroup differences while minimizing intragroup variation and filtering out irrelevant information. Scoring plots for each group were generated (Figure 8A), enabling effective differentiation among samples across different groups. The reliability assessment of the model established for this sample (Figure 8B) indicated that Q^2^ and R^2^Y were close to 1, demonstrating that the model was reliable.

Differential metabolites were screened based on VIP > 1, fold change ≥ 2, and fold change ≤ 0.5, yielding 273 differential metabolites. These were visualized using a volcano plot (Figure 8C). Among the samples, 92 differentially expressed metabolites showed significant upregulation, while 87 exhibited significant downregulation. Knockout of Pir7b reduced syringolin A‐induced resistance to rice blast disease (Figure 7), indicating that numerous blast‐resistant genes and metabolites were suppressed in pir7bA samples. Therefore, downregulated substances underwent enrichment analysis and heatmap analysis of their expression levels. The heatmap of binding content displayed the top 20 differentially expressed metabolites with VIP values greater than 1 (Figure 8D). KEGG enrichment analysis results indicated (Figure 8E) enrichment in pathways related to alpha‐linolenic acid metabolism, cutin, suberin, and wax biosynthesis, terpenoid backbone biosynthesis, purine metabolism, and phenylpropanoid biosynthesis. Among these, alpha‐Linolenic acid metabolism, Cutin, suberine and wax biosynthesis, and Phenylpropanoid biosynthesis are associated with ROS activity. These pathways play a crucial role in maintaining ROS homeostasis (Mittler et al. 2022). Crucially, the VIP values for the differential substances MEDL02242_Carvacrol, MW0056883_Colfosceril palmitate, and MEDN0204_Pyruvic acid all exceeded 1.3 (Figure 8F). Moreover, previous studies have demonstrated that these three pathways exhibit synergistic effects with the PTI template, playing a crucial role (Boller and He 2009; Chisholm et al. 2006).

## 4 Discussion

Plants are generally considered to lack the adaptive immunity of animals, which relies on antibodies, but their innate immunity is similar to that of animals. Under the stimulation of different PAMPs, plants activate their first layer of innate immunity to defend against pathogen invasion. Interestingly, non‐host pathogens themselves cannot infect non‐host plants, but they can induce non‐host plant immune resistance by PAMPs (Ingle et al. 2006; Uma et al. 2011). Although this phenomenon has long been discovered, there is currently no clear model to reveal its complex induction mechanism. This study aims to uncover the phenomenon that a peptide elicitor secreted by non‐host pathogens can initiate immune resistance of rice, and further explore resistance genes with different activation mechanisms.

It was successively reported that NAC transcription factors in rice have been found to be directly related to blast resistance. OsNAC19 may function as a component of the MeJA‐mediated signaling pathway and play a role in response to *M. oryzae* infection (Lin et al. 2007). ONAC0066 positively regulates rice resistance to blast and bacterial blight by modulating the ABA signaling pathway and the accumulation of sugars and amino acids (Liu et al. 2018). ONAC083 negatively regulates rice immune response to *M. oryzae* by activating the transcription of *OsRFPH2‐6* (Bi et al. 2023). It is noteworthy that the NAC transcription factors involved in the immune process were induced by *M. oryzae*, whereas this study found that *OsNAC78* is not induced by *M. oryzae*, but by syringolin A secreted by the non‐host pathogen *P. syringae* (Supporting Information S1: Figure 5A; Figure 5). This indicates that the mechanisms by which the NAC family is induced during the fight against pathogens are diverse, leading to differing levels of resistance strength. It also reflects that there are significant differences in the receptor PRR response mechanisms corresponding to PAMPs of host and non‐host pathogens. Further in‐depth research on this is of great significance.

The binding target Pir7b of OsNAC78 was captured through high‐resolution analysis using 10 × Genomics' single‐cell RNA sequencing method technology (Xie et al. 2020). Although it was previously reported that the defense‐related gene *Pir7b* was induced by syringolin, and syringolin treatment of rice plants could increase resistance to *M. oryzae* (Wäspi et al. 2007). However, this evidence does not directly indicate Pir7b has the function of resisting rice blast disease. This study involves inoculating transgenic rice with knocked‐out or overexpressed *Pir7b* gene respectively to assess their resistance to blast disease. Regardless of punch or spray inoculation, the results showed that Pir7b consistently confers the blast resistance in rice (Figure 3). *Pir7b*, like *OsNAC78*, is not induced by *M. oryzae*, but by non‐host pathogens (Supporting Information S1: Figure 5; Figure 5). This reveals that the transcription factor OsNAC78 regulates the expression of Pir7b in response to stress from non‐host pathogens. Compared to host resistance, the research of non‐host resistance is generally more confused, often due to the lack of intraspecific polymorphism. Fortunately, over the past two decades, significant progress has been made in the study of non‐host resistance, revealing that the molecular mechanisms of non‐host resistance involve PTI and effector‐triggered immunity (Thordal‐Christensen 2003; Wu et al. 2023). Here, it was revealed that *P. syringae* induces rice resistance to blast fungus, and we speculate that the immune response triggered by the OsNAC78‐Pir7b module may be of PTI type.

The blast resistance is regulated with multiple ways in rice (Jin et al. 2024; Qi et al. 2024; Zhao et al. 2025). In this study, after treatment with syringin A, differentially synthesized metabolites from *pir7b* mutants and wild‐type varieties were enriched in pathways such as alpha‐linolenic acid metabolism, cutin, suberine and wax biosynthesis. Among them, alpha‐linolenic acid metabolism is closely related to disease resistance(Li et al. 2025a). MEDN0204_Pyruvic acid participates in ROS homeostasis maintenance after PTI‐mediated oxidative burst in the antioxidant system pathway (Dangl and Jones 2001). MEDL02026_4‐Hydroxy‐3‐methoxycinnamaldehyde is located in the Phenylpropanoid biosynthesis pathway, a downstream secondary metabolic pathway of PTIs, which is activated in the PTI defense response (Boller and He 2009; Chisholm et al. 2006). These differentially metabolites were enriched in pathways associated with the PTI system and play an important role. In addition, pathways related to the synthesis of defense signaling molecules, such as phenylpropanoid biosynthesis and purine metabolism, were also enriched (Li et al. 2025b). This implies they may be involved in the resistance defense response against rice blast disease.

**Figure 9:**
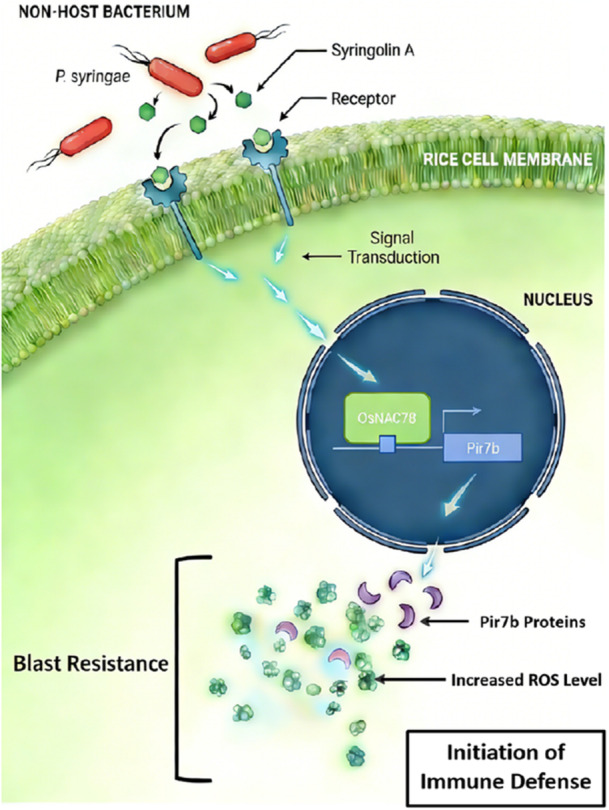
The working model of syringolin A induces blast resistance mediated by OsNAC78‐Pir7b module in rice. [Color figure can be viewed at wileyonlinelibrary.com]

The non‐host resistance is broad‐spectrum and persistent. Generally speaking, this study provides an example of the secretion of non‐host pathogenic bacteria inducing immunity in rice. After sensing syringin A, rice cells initiate an immune response; following signal transduction, the transcription factor OsNAC78 is induced, directly regulating the expression of the downstream defense gene *Pir7b*, thereby generating resistance to blast disease (Figure 9). Currently, conducting in‐depth research on the molecular mechanisms of non‐host resistance is of great significance, especially for diseases that lack effective resistance sources. For example, diseases such as rice false smut and citrus Huanglongbing, which are increasingly rampant, urgently need effective resistance genes to control outbreaks. Coincidentally, non‐host resistance genes may serve as a breakthrough point for solving this problem. Therefore, non‐host resistance genes, as important potential disease‐resistant resources in breeding, are one of the current and future hotspots in plant immunity research.

## Conflicts of Interest

The authors declare no conflicts of interest.

## Supporting information


**Supporting Information S1: Figure 1.** OsNAC78 positively regulates resistance to rice blast. **Supporting Information S1: Figure 2.** Knockout site and overexpression of Pir7b in Nipponbare background. **Supporting Information S1: Figure 3.** Detection of ROS in Pir7b transgenic rice. **Supporting Information S1: Figure 4.** Phylogenetic tree of Pir7b related proteins. **Supporting Information S1: Figure 5.** The expression of OsNAC78 and Pir7b was not significant induced by M.oryzae.

**Supporting Information S2: Table 1.** Primer used in this study.

## References

[pce70500-bib-0001] Bi, Y. , H. Wang , X. Yuan , Y. Yan , D. Li , and F. Song . 2023. “The NAC Transcription Factor ONAC083 Negatively Regulates Rice Immunity Against *Magnaporthe oryzae* by Directly Activating Transcription of the RING‐H2 Gene OsRFPH2‐6.” Journal of Integrative Plant Biology 65, no. 3: 854–875. 10.1111/jipb.13399. 36308720

[pce70500-bib-0002] Boller, T. , and S. Y. He . 2009. “Innate Immunity in Plants: An Arms Race Between Pattern Recognition Receptors in Plants and Effectors in Microbial Pathogens.” Science 324, no. 5928: 742–744. 10.1126/science.1171647. 19423812 PMC2729760

[pce70500-bib-0003] Boutrot, F. , and C. Zipfel . 2017. “Function, Discovery, and Exploitation of Plant Pattern Recognition Receptors for Broad‐Spectrum Disease Resistance.” Annual Review of Phytopathology 55: 257–286. 10.1146/annurev-phyto-080614-120106. 28617654

[pce70500-bib-0004] Cai, J. , S. Panda , and Y. Kazachkova , et al. 2024. “A NAC Triad Modulates Plant Immunity by Negatively Regulating N‐Hydroxy Pipecolic Acid Biosynthesis.” Nature Communications 15, no. 1: 7212. 10.1038/s41467-024-51515-2. PMC11341717 39174537

[pce70500-bib-0005] Chang, Y. , Y. Fang , and J. Liu , et al. 2024. “Stress‐Induced Nuclear Translocation of ONAC023 Improves Drought and Heat Tolerance Through Multiple Processes in Rice.” Nature Communications 15, no. 11: 5877. 10.1038/s41467-024-50229-9. PMC11245485 38997294

[pce70500-bib-0006] Chisholm, S. T. , G. Coaker , B. Day , and B. J. Staskawicz . 2006. “Host‐Microbe Interactions: Shaping the Evolution of the Plant Immune Response.” Cell 124, no. 4: 803–814. 10.1016/j.cell.2006.02.008. 16497589

[pce70500-bib-0007] Couto, D. , and C. Zipfel . 2016. “Regulation of Pattern Recognition Receptor Signalling in Plants.” Nature Reviews Immunology 16, no. 9: 537–552. 10.1038/nri.2016.77. 27477127

[pce70500-bib-0008] Dangl, J. L. , and J. D. G. Jones . 2001. “Plant Pathogens and Integrated Defence Responses to Infection.” Nature 411, no. 6839: 826–833. 10.1038/35081161. 11459065

[pce70500-bib-0009] Han, K. , Y. Zhao , Y. Sun , and Y. Li . 2023. “NACs,Generalist in Plant Life.” Plant Biotechnology Journal 21, no. 12: 2433–2457. 10.1111/pbi.14161. 37623750 PMC10651149

[pce70500-bib-0010] Hassa, P. , J. Granado , E. Freydl , U. Wäspi , and R. Dudler . 2000. “Syringolin‐Mediated Activation of the Pir7b Esterase Gene in Rice Cells is Suppressed by Phosphatase Inhibitors.” Molecular Plant‐Microbe Interactions® 13, no. 3: 342–346. 10.1094/MPMI.2000.13.3.342. 10707360

[pce70500-bib-0011] Ingle, R. A. , M. Carstens , and K. J. Denby . 2006. “PAMP Recognition and the Plant‐Pathogen Arms Race.” BioEssays 28, no. 9: 880–889. 10.1002/bies.20457. 16937346

[pce70500-bib-0012] Jin, B. J. , H. J. Chun , C. W. Choi , et al. 2024. “Host‐Induced Gene Silencing is a Promising Biological Tool to Characterize the Pathogenicity of *Magnaporthe oryzae* and Control Fungal Disease in Rice.” Plant, Cell & Environment 47, no. 1: 319–336. 10.1111/pce.14721. 37700662

[pce70500-bib-0013] Jin, X. , J. Ren , E. Nevo , X. Yin , D. Sun , and J. Peng . 2017. “Divergent Evolutionary Patterns of NAC Transcription Factors are Associated With Diversification and Gene Duplications in Angiosperm.” Frontiers in Plant Science 8: 1156. 10.3389/fpls.2017.01156. 28713414 PMC5492850

[pce70500-bib-0014] Kadota, Y. , J. Sklenar , P. Derbyshire , et al. 2014. “Direct Regulation of the NADPH Oxidase RBOHD by the PRR‐Associated Kinase BIK1 During Plant Immunity.” Molecular Cell 54, no. 1: 43–55. 10.1016/j.molcel.2014.02.021. 24630626

[pce70500-bib-0015] Lee, H. A. , S. Y. Kim , S. K. Oh , et al. 2014. “Multiple Recognition of RXLR Effectors is Associated With Nonhost Resistance of Pepper Against Phytophthora infestans.” New Phytologist 203, no. 3: 926–938. 10.1111/nph.12861. 24889686 PMC4143959

[pce70500-bib-0016] Li, F. , J. Wu , and L. Zhang , et al. 2025b. “Elucidating the Mechanism of Resistance to Anthracnose in Litchi Leaves Through Transcriptome Analysis.” BMC Plant Biology 25, no. 1: 384. 10.1186/s12870-025-06382-4. 40133872 PMC11938760

[pce70500-bib-0017] Li, G. , X. Meng , R. Wang , et al. 2012. “Dual‐Level Regulation of ACC Synthase Activity by MPK3/MPK6 Cascade and Its Downstream WRKY Transcription Factor During Ethylene Induction in Arabidopsis.” PLoS Genetics 8, no. 6: e1002767. 10.1371/journal.pgen.1002767. 22761583 PMC3386168

[pce70500-bib-0018] Li, W. , Z. Zhu , M. Chern , et al. 2017. “A Natural Allele of a Transcription Factor in Rice Confers Broad‐Spectrum Blast Resistance.” Cell 170, no. 1: 114–126.e15. 10.1016/j.cell.2017.06.008. 28666113

[pce70500-bib-0019] Li, X. , Y. Chang , S. Ma , J. Shen , H. Hu , and L. Xiong . 2019. “Genome‐Wide Identification of SNAC1‐Targeted Genes Involved in Drought Response in Rice.” Frontiers in Plant Science 10: 982. 10.3389/fpls.2019.00982. 31402926 PMC6677020

[pce70500-bib-0020] Li, Y. , L. Govta , Y. C. Sung , G. Coaker , and T. Fahima . 2025a. “The Spectrum of Diverse Disease‐Resistance Genes Cloned and Characterized in the Triticeae Tribe.” Annual Review of Phytopathology 63, no. 1: 175–200. 10.1146/annurev-phyto-121323-031121. 40523106

[pce70500-bib-0021] Lin, R. , W. Zhao , X. Meng , M. Wang , and Y. Peng . 2007. “Rice Gene OsNAC19 Encodes a Novel NAC‐Domain Transcription Factor and Responds to Infection by Magnaporthe grisea.” Plant Science 172, no. 1: 120–130. 10.1016/j.plantsci.2006.07.019.

[pce70500-bib-0022] Liu, J. , Y. Qiao , C. Li , and B. Hou . 2023. “The NAC Transcription Factors Play Core Roles in Flowering and Ripening Fundamental to Fruit Yield and Quality.” Frontiers in Plant Science 14: 1095967. 10.3389/fpls.2023.1095967. 36909440 PMC9996081

[pce70500-bib-0023] Liu, Q. , S. Yan , W. Huang , et al. 2018. “NAC Transcription Factor ONAC066 Positively Regulates Disease Resistance by Suppressing the ABA Signaling Pathway in Rice.” Plant Molecular Biology 98: 289–302. 10.1007/s11103-018-0768-z. 30387038

[pce70500-bib-0024] Lu, L. , J. Zhang , X. Zheng , et al. 2024. “OsMPK12 Positively Regulates Rice Blast Resistance via OsEDC4‐Mediated Transcriptional Regulation of Immune‐Related Genes.” Plant, Cell & Environment 47, no. 10: 3712–3731. 10.1111/pce.14955. 38770581

[pce70500-bib-0025] Luo, Q. , W. W. Han , Y. H. Zhou , Y. Yao , and Z. S. Li . 2008. “The 3D Structure of the Defense‐Related Rice Protein Pir7b Predicted by Homology Modeling and Ligand Binding Studies.” Journal of Molecular Modeling 14, no. 7: 559–569. 10.1007/s00894-008-0310-3. 18449577

[pce70500-bib-0026] Mao, C. , J. He , L. Liu , et al. 2020a. “OsNAC2 Integrates Auxin and Cytokinin Pathways to Modulate Rice Root Development.” Plant Biotechnology Journal 18, no. 2: 429–442. 10.1111/pbi.13209. 31389120 PMC6953191

[pce70500-bib-0027] Mao, H. , S. Li , Z. Wang , et al. 2020b. “Regulatory Changes in TaSNAC8‐6A Are Associated With Drought Tolerance in Wheat Seedlings.” Plant Biotechnology Journal 18, no. 4: 1078–1092. 10.1111/pbi.13277. 31617659 PMC7061879

[pce70500-bib-0028] Mittler, R. , S. I. Zandalinas , Y. Fichman , and F. Van Breusegem . 2022. “Reactive Oxygen Species Signalling in Plant Stress Responses.” Nature Reviews Molecular Cell Biology 23, no. 10: 663–679. 10.1038/s41580-022-00499-2. 35760900

[pce70500-bib-0029] Mohanta, T. K. , D. Yadav , A. Khan , et al. 2020. “Genomics, Molecular and Evolutionary Perspective of NAC Transcription Factors.” PLoS One 15, no. 4: e0231425. 10.1371/journal.pone.0231425. 32275733 PMC7147800

[pce70500-bib-0030] Olsen, A. N. , H. A. Ernst , L. L. Leggio , and K. Skriver . 2005. “NAC Transcription Factors: Structurally Distinct, Functionally Diverse.” Trends in Plant Science 10, no. 2: 79–87. 10.1016/j.tplants.2004.12.010. 15708345

[pce70500-bib-0031] Park, C. H. , S. Chen , G. Shirsekar , et al. 2012. “The *Magnaporthe oryzae* Effector Avrpiz‐T Targets the RING E3 Ubiquitin Ligase APIP6 to Suppress Pathogen‐Associated Molecular Pattern‐Triggered Immunity in Rice.” Plant Cell 24, no. 11: 4748–4762. 10.1105/tpc.112.105429. 23204406 PMC3531864

[pce70500-bib-0032] Qi, Z. , X. Meng , M. Xu , et al. 2024. “A Novel Pik Allele Confers Extended Resistance to Rice Blast.” Plant, Cell & Environment 47, no. 12: 4800–4814. 10.1111/pce.15072. 39087779

[pce70500-bib-0033] Qu, S. , G. Liu , B. Zhou , et al. 2006. “The Broad‐Spectrum Blast Resistance Gene Pi9 Encodes a Nucleotide‐Binding Site‐Leucine‐Rich Repeat Protein and is a Member of a Multigene Family in Rice.” Genetics 172, no. 3: 1901–1914. 10.1534/genetics.105.044891. 16387888 PMC1456263

[pce70500-bib-0034] Reimmann, C. , C. Hofmann , F. Mauch , and R. Dudler . 1995. “Characterization of a Rice Gene Induced by Pseudomonas syringae pv. Syringae: Requirement for the Bacterial lemA Gene Function.” Physiological and Molecular Plant Pathology 46, no. 1: 71–81. 10.1006/pmpp.1995.1006.

[pce70500-bib-0035] Rui, L. , S. Q. Yang , X. H. Zhou , and W. Wang . 2025. “The Important Role of Chloroplasts in Plant Immunity.” Plant Communications 6, no. 8: 101420. 10.1016/j.xplc.2025.101420. 40534128 PMC12365846

[pce70500-bib-0036] Thordal‐Christensen, H. 2003. “Fresh Insights into Processes of Nonhost Resistance.” Current Opinion in Plant Biology 6, no. 4: 351–357. 10.1016/S1369-5266(03)00063-3. 12873530

[pce70500-bib-0037] Tian, W. , C. Hou , Z. Ren , et al. 2019. “A Calmodulin‐Gated Calcium Channel Links Pathogen Patterns to Plant Immunity.” Nature 572: 131–135. 10.1038/s41586-019-1413-y. 31316205

[pce70500-bib-0038] Uma, B. , T. Swaroopa Rani , and A. R. Podile . 2011. “Warriors at the Gate That Never Sleep: Non‐Host Resistance in Plants.” Journal of Plant Physiology 168, no. 18: 2141–2152. 10.1016/j.jplph.2011.09.005. 22001579

[pce70500-bib-0039] Vega‐Arreguín, J. C. , A. Jalloh , J. I. Bos , and P. Moffett . 2014. “Recognition of an Avr3a Homologue Plays a Major Role in Mediating Nonhost Resistance to Phytophthora capsici in Nicotiana Species.” Molecular Plant‐Microbe Interactions® 27, no. 8:7: 770–780. 10.1094/MPMI-01-14-0014-R. 24725207

[pce70500-bib-0040] Wang, H. , Y. Bi , Y. Yan , et al. 2024. “A NAC Transcription Factor MNAC3‐Centered Regulatory Network Negatively Modulates Rice Immunity Against Blast Disease.” Journal of Integrative Plant Biology 66, no. 9: 2017–2041. 10.1111/jipb.13727. 38953747

[pce70500-bib-0041] Wang, J. , C. Zheng , and X. Shao , et al. 2020. “Transcriptomic and Genetic Approaches Reveal an Essential Role of the NAC Transcription Factor SlNAP1 in the Growth and Defense Response of Tomato.” Horticulture Research 7, no. 1: 209. 10.1038/s41438-020-00442-6. 33361767 PMC7759572

[pce70500-bib-0042] Wäspi, U. , D. Blanc , T. Winkler , P. Rüedi , and R. Dudler . 1998. “Syringolin, a Novel Peptide Elicitor From Pseudomonas syringae pv. syringae That Induces Resistance to Pyricularia oryzae in Rice.” Molecular Plant‐Microbe Interactions® 11, no. 8: 727–733. 10.1094/MPMI.1998.11.8.727.

[pce70500-bib-0043] Wu, Y. , W. Sexton , B. Yang , and S. Xiao . 2023. “Genetic Approaches to Dissect Plant Nonhost Resistance Mechanisms.” Molecular Plant Pathology 24, no. 3: 272–283. 10.1111/mpp.13290. 36617319 PMC9923397

[pce70500-bib-0044] Xiang, Y. , N. Tang , H. Du , H. Ye , and L. Xiong . 2008. “Characterization of OsbZIP23 as a Key Player of the Basic Leucine Zipper Transcription Factor Family for Conferring Abscisic Acid Sensitivity and Salinity and Drought Tolerance in Rice.” Plant Physiology 148, no. 4: 1938–1952. 10.1104/pp.108.128199. 18931143 PMC2593664

[pce70500-bib-0045] Xie, Y. , S. Jiang , L. Li , et al. 2020. “Single‐Cell RNA Sequencing Efficiently Predicts Transcription Factor Targets in Plants.” Frontiers in Plant Science 11: 603302. 10.3389/fpls.2020.603302. 33424903 PMC7793804

[pce70500-bib-0046] Xing, H. L. , L. Dong , and Z. P. Wang , et al. 2014. “A CRISPR/Cas9 Toolkit for Multiplex Genome Editing in Plants.” BMC Plant Biology 14: 327. 10.1186/s12870-014-0327-y. 25432517 PMC4262988

[pce70500-bib-0047] Xiong, H. , H. He , Y. Chang , et al. 2025. “Multiple Roles of NAC Transcription Factors in Plant Development and Stress Responses.” Journal of Integrative Plant Biology 67, no. 3: 510–538. 10.1111/jipb.13854. 39950532

[pce70500-bib-0048] Yang, L. , L. Xu , J. Guo , et al. 2024. “SNAC1‐OsERF103‐OsSDG705 Module Mediates Drought Response in Rice.” New Phytologist 241, no. 6: 2480–2494. 10.1111/nph.19552. 38296835

[pce70500-bib-0049] Yoo, S. D. , Y. H. Cho , and J. Sheen . 2007. “Arabidopsis Mesophyll Protoplasts: A Versatile Cell System for Transient Gene Expression Analysis.” Nature Protocols 2: 1565–1572. 10.1038/nprot.2007.199. 17585298

[pce70500-bib-0050] Yu, X. , Y. Xie , L. Wang , et al. 2024b. “Transcription Factor NAC78 Cooperates With NAC78 Interacting Protein 6 to Confer Drought Tolerance in Rice.” Plant Physiology 196, no. 2: 1642–1658. 10.1093/plphys/kiae395. 39082752

[pce70500-bib-0051] Yu, X. Q. , H. Q. Niu , C. Liu , H. L. Wang , W. Yin , and X. Xia . 2024a. “PTI‐ETI Synergistic Signal Mechanisms in Plant Immunity.” Plant Biotechnology Journal 22, no. 8: 2113–2128. 10.1111/pbi.14332. 38470397 PMC11258992

[pce70500-bib-0052] Zhang, Y. , J. Su , S. Duan , et al. 2011. “A Highly Efficient Rice Green Tissue Protoplast System for Transient Gene Expression and Studying Light/Chloroplast ‐Related Processes.” Plant Methods 7: 30. 10.1186/1746-4811-7-30. 21961694 PMC3203094

[pce70500-bib-0053] Zhao, T. , P. Tang , R. Zuo , et al. 2025. “OsPIL1 Differentially Modulates Rice Blast Resistance Through Integrating Light or Darkness During *Magnaporthe oryzae* Infection.” Plant, Cell & Environment 48, no. 12: 8839–8859. 10.1111/pce.70168. 40916382

