# Supplementary material for: A Non‐Host Pathogen Elicitor Induces Blast Resistance Mediated by OsNAC78‐Pir7b Module in Rice
Source: Plant Cell Environ. 2026 Mar 29;49(8):4894–906. doi: 10.1111/pce.70500 (PMC13353671; doi:10.1111/pce.70500)
Supplement: Supplementary file 1 — Supporting Information S1: Figure 1. OsNAC78 positively regulates resistance to rice blast. Supporting Information S1: Figure 2. Knockout site and overexpression of Pir7b in Nipponbare background. Supporting Information S1: Figure 3. Detection of ROS in Pir7b transgenic rice. Supporting Information S1: Figure 4. Phylogenetic tree of Pir7b related proteins. Supporting Information S1: Figure 5. The expression of OsNAC78 and Pir7b was not significant induced by M.oryzae. [file PCE-49-4894-s001.pptx]

## Slide 1
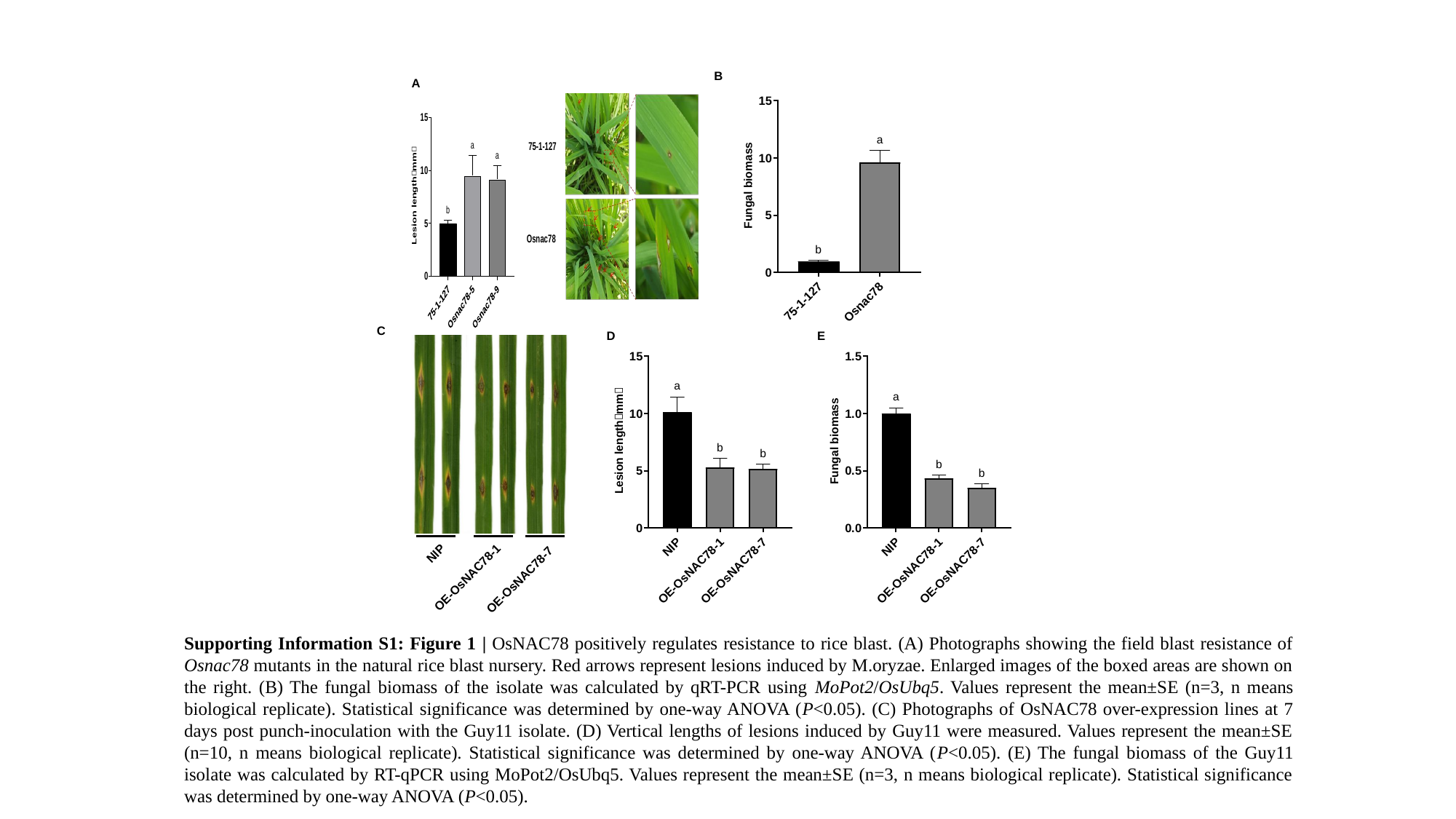

Supporting Information S1: Figure 1 | OsNAC78 positively regulates resistance to rice blast. (A) Photographs showing the field blast resistance of Osnac78 mutants in the natural rice blast nursery. Red arrows represent lesions induced by M.oryzae. Enlarged images of the boxed areas are shown on the right. (B) The fungal biomass of the isolate was calculated by qRT-PCR using MoPot2/OsUbq5. Values represent the mean±SE (n=3, n means biological replicate). Statistical significance was determined by one-way ANOVA (P<0.05). (C) Photographs of OsNAC78 over-expression lines at 7 days post punch-inoculation with the Guy11 isolate. (D) Vertical lengths of lesions induced by Guy11 were measured. Values represent the mean±SE (n=10, n means biological replicate). Statistical significance was determined by one-way ANOVA (P<0.05). (E) The fungal biomass of the Guy11 isolate was calculated by RT-qPCR using MoPot2/OsUbq5. Values represent the mean±SE (n=3, n means biological replicate). Statistical significance was determined by one-way ANOVA (P<0.05).

## Slide 2
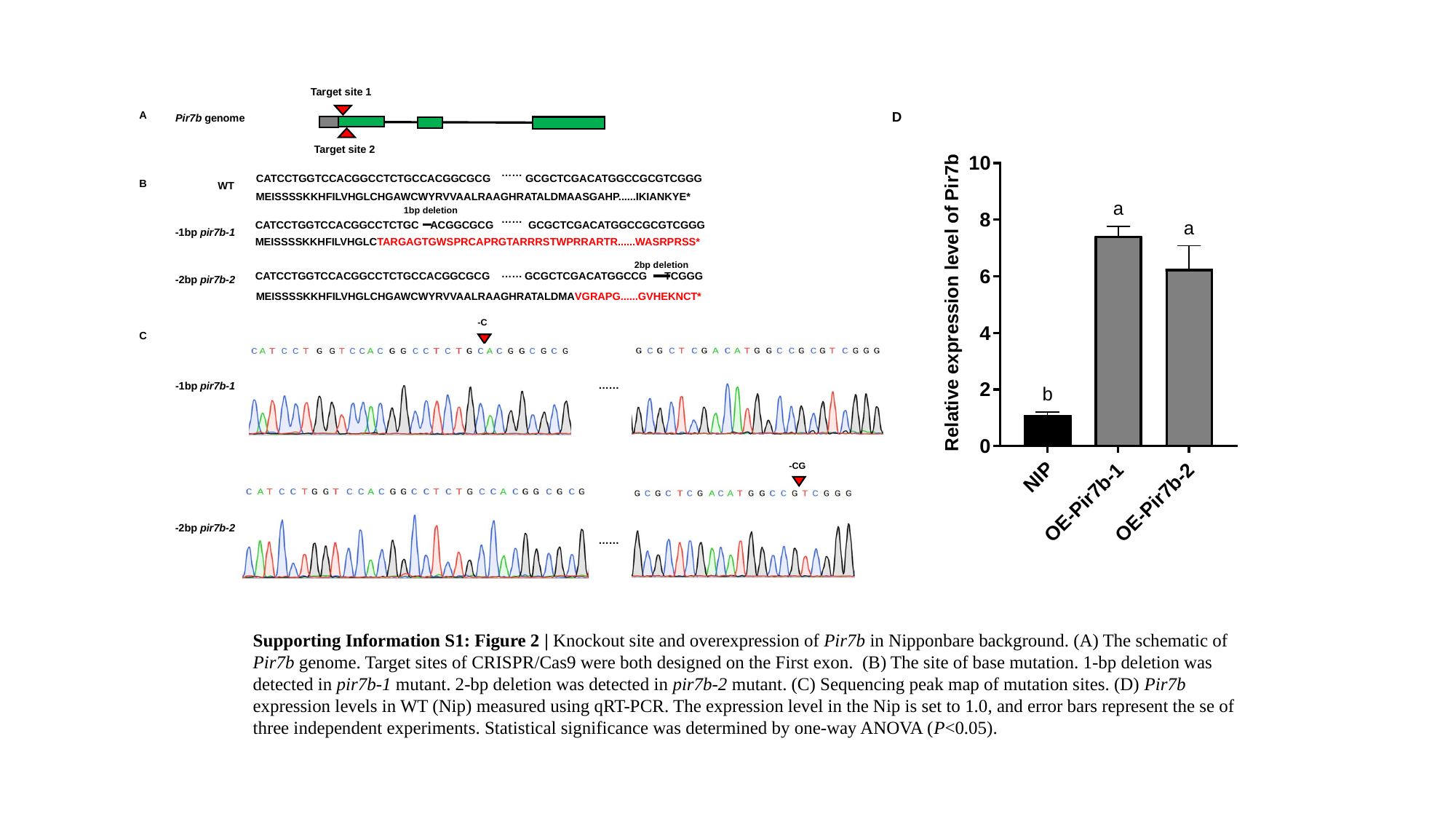

Target site 1
Target site 2
A
Pir7b genome
……
CATCCTGGTCCACGGCCTCTGCCACGGCGCG GCGCTCGACATGGCCGCGTCGGG
B
WT
MEISSSSKKHFILVHGLCHGAWCWYRVVAALRAAGHRATALDMAASGAHP......IKIANKYE*
1bp deletion
……
CATCCTGGTCCACGGCCTCTGC ACGGCGCG GCGCTCGACATGGCCGCGTCGGG
-1bp pir7b-1
MEISSSSKKHFILVHGLCTARGAGTGWSPRCAPRGTARRRSTWPRRARTR......WASRPRSS*
2bp deletion
……
CATCCTGGTCCACGGCCTCTGCCACGGCGCG GCGCTCGACATGGCCG TCGGG
-2bp pir7b-2
MEISSSSKKHFILVHGLCHGAWCWYRVVAALRAAGHRATALDMAVGRAPG......GVHEKNCT*
-C
……
C
-1bp pir7b-1
-CG
……
-2bp pir7b-2
……
D
Supporting Information S1: Figure 2 | Knockout site and overexpression of Pir7b in Nipponbare background. (A) The schematic of Pir7b genome. Target sites of CRISPR/Cas9 were both designed on the First exon. (B) The site of base mutation. 1-bp deletion was detected in pir7b-1 mutant. 2-bp deletion was detected in pir7b-2 mutant. (C) Sequencing peak map of mutation sites. (D) Pir7b expression levels in WT (Nip) measured using qRT-PCR. The expression level in the Nip is set to 1.0, and error bars represent the se of three independent experiments. Statistical significance was determined by one-way ANOVA (P<0.05).

## Slide 3
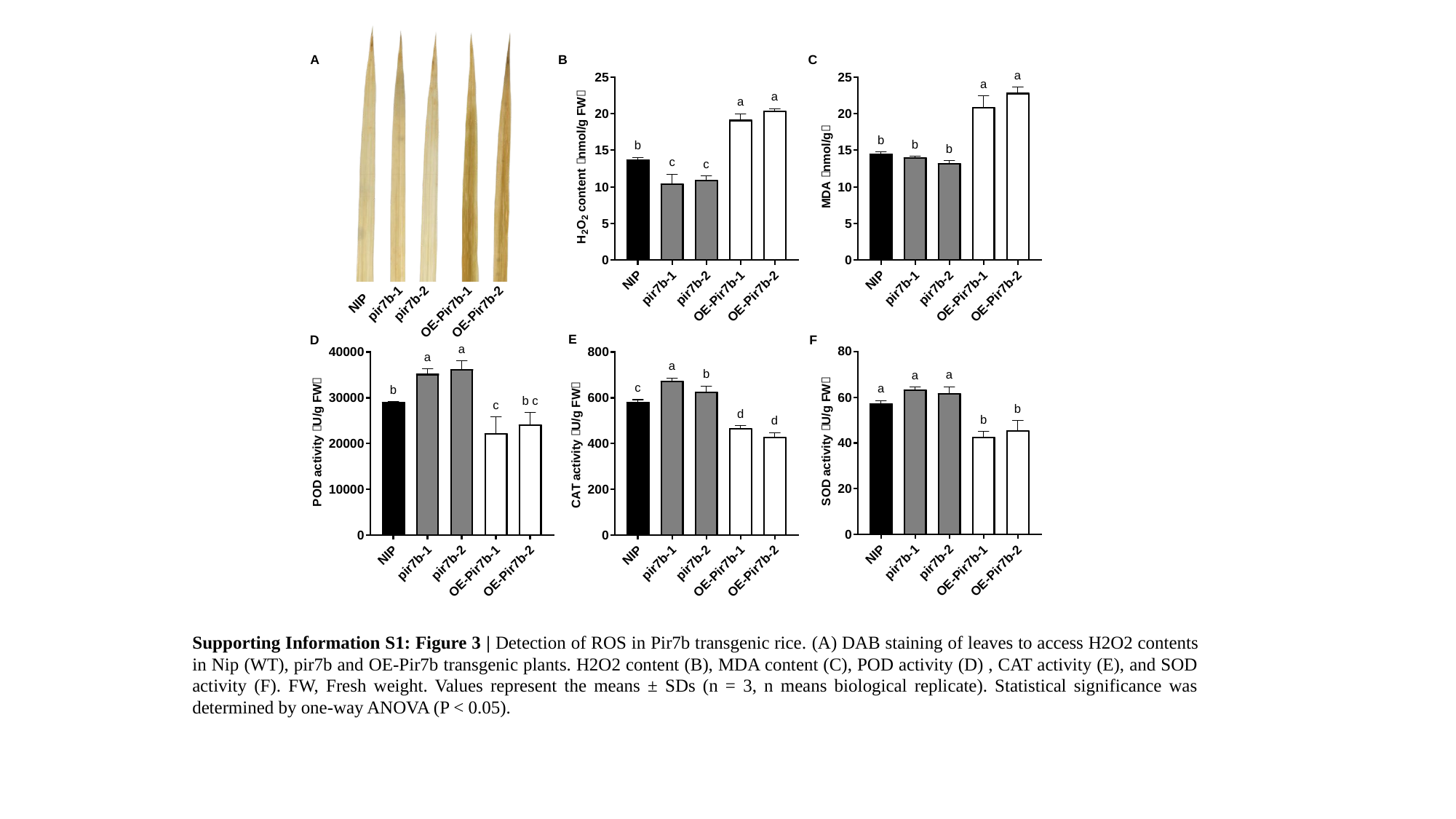

Supporting Information S1: Figure 3 | Detection of ROS in Pir7b transgenic rice. (A) DAB staining of leaves to access H2O2 contents in Nip (WT), pir7b and OE-Pir7b transgenic plants. H2O2 content (B), MDA content (C), POD activity (D) , CAT activity (E), and SOD activity (F). FW, Fresh weight. Values represent the means ± SDs (n = 3, n means biological replicate). Statistical significance was determined by one-way ANOVA (P < 0.05).

## Slide 4
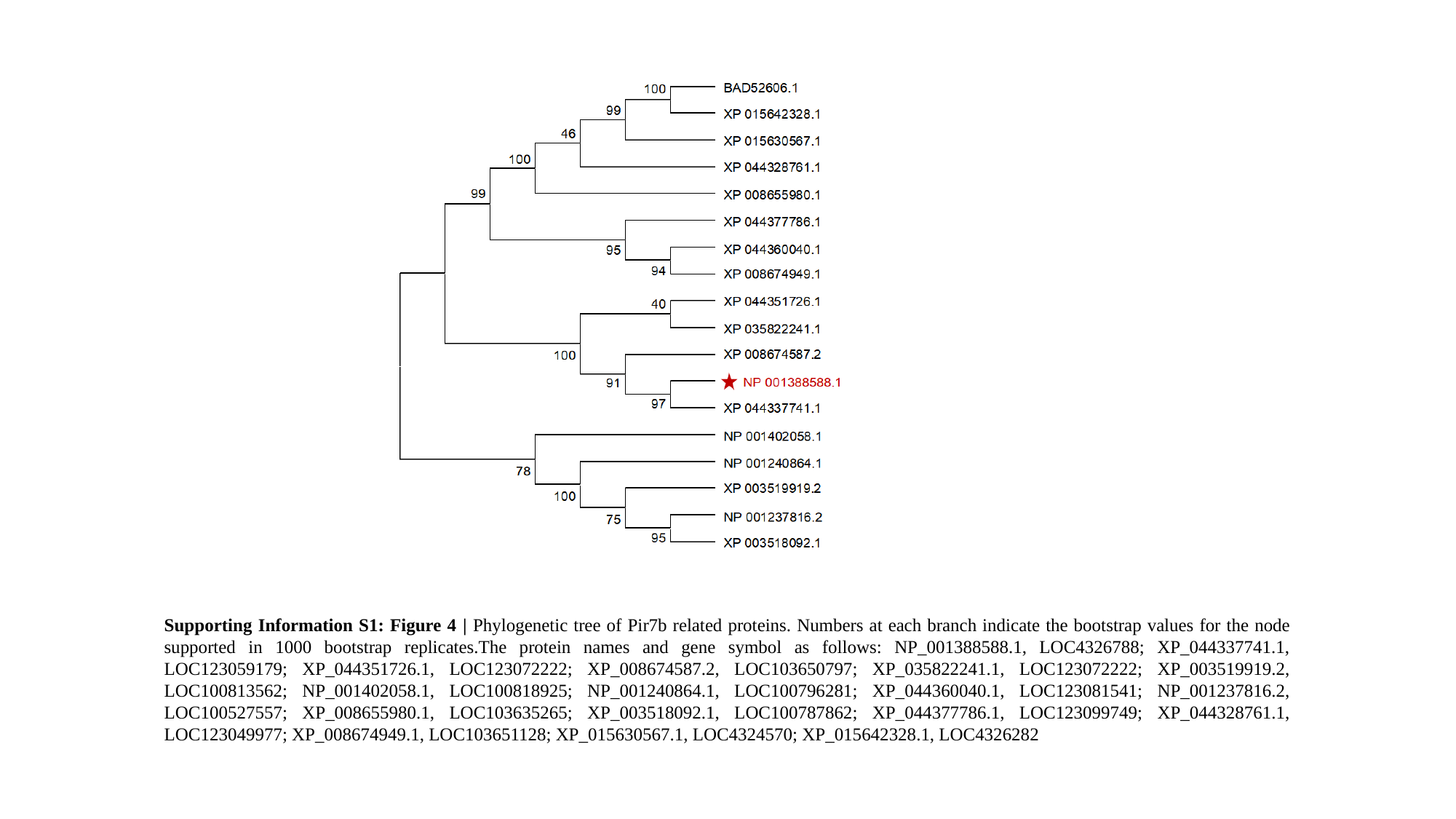

Supporting Information S1: Figure 4 | Phylogenetic tree of Pir7b related proteins. Numbers at each branch indicate the bootstrap values for the node supported in 1000 bootstrap replicates.The protein names and gene symbol as follows: NP_001388588.1, LOC4326788; XP_044337741.1, LOC123059179; XP_044351726.1, LOC123072222; XP_008674587.2, LOC103650797; XP_035822241.1, LOC123072222; XP_003519919.2, LOC100813562; NP_001402058.1, LOC100818925; NP_001240864.1, LOC100796281; XP_044360040.1, LOC123081541; NP_001237816.2, LOC100527557; XP_008655980.1, LOC103635265; XP_003518092.1, LOC100787862; XP_044377786.1, LOC123099749; XP_044328761.1, LOC123049977; XP_008674949.1, LOC103651128; XP_015630567.1, LOC4324570; XP_015642328.1, LOC4326282

## Slide 5
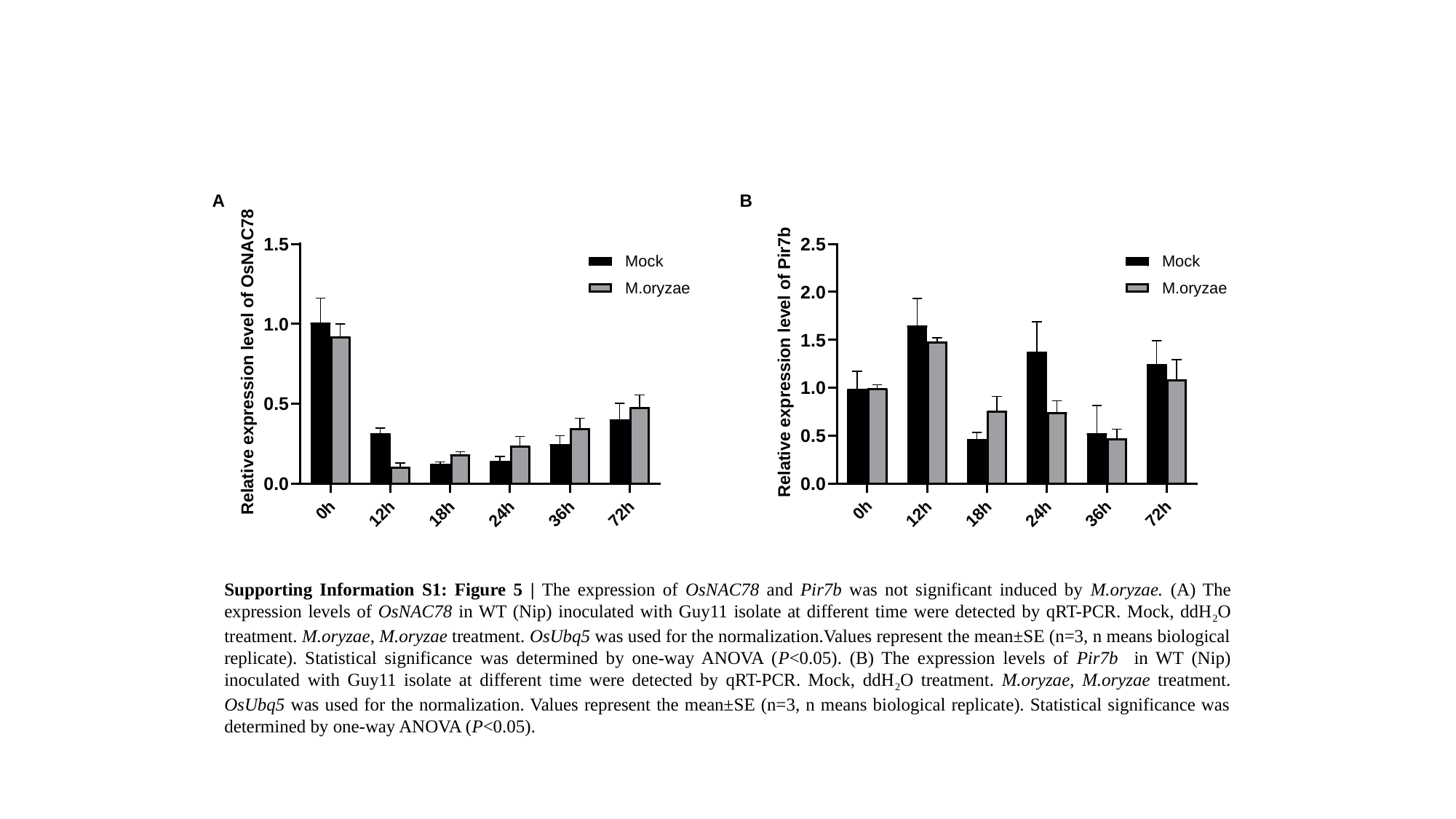

Supporting Information S1: Figure 5 | The expression of OsNAC78 and Pir7b was not significant induced by M.oryzae. (A) The expression levels of OsNAC78 in WT (Nip) inoculated with Guy11 isolate at different time were detected by qRT-PCR. Mock, ddH2O treatment. M.oryzae, M.oryzae treatment. OsUbq5 was used for the normalization.Values represent the mean±SE (n=3, n means biological replicate). Statistical significance was determined by one-way ANOVA (P<0.05). (B) The expression levels of Pir7b in WT (Nip) inoculated with Guy11 isolate at different time were detected by qRT-PCR. Mock, ddH2O treatment. M.oryzae, M.oryzae treatment. OsUbq5 was used for the normalization. Values represent the mean±SE (n=3, n means biological replicate). Statistical significance was determined by one-way ANOVA (P<0.05).
